# DNA Methylation Shapes Seed‐Borne Microbiome and Proteome Responses During Early Maize‐Beneficial Bacteria Interactions

**DOI:** 10.1111/pce.70552

**Published:** 2026-04-22

**Authors:** Pedro Igor Zocateli, Geovanna Vitória Olímpio, Felipe Astolpho de Almeida, Letícia Oliveira da Rocha, Diego Vinicius de Sena Martins, Sara Sangi, Fábio Lopes Olivares, Elyabe Monteiro de Matos, Juliana Siqueira, Lyderson Facio Viccini, Roberta Pena da Paschoa, Vanildo Silveira, Clícia Grativol

**Affiliations:** ^1^ Laboratório de Química e Função de Proteínas e Peptídeos, Centro de Biociências e Biotecnologia Universidade Estadual do Norte Fluminense Darcy Ribeiro Rio de Janeiro Campos dos Goytacazes Brazil; ^2^ Laboratório de Biologia Celular e Tecidual, Centro de Biociências e Biotecnologia Universidade Estadual do Norte Fluminense Darcy Ribeiro Rio de Janeiro Campos dos Goytacazes Brazil; ^3^ Laboratório de Genética e Biotecnologia, Departamento de Biologia Universidade Federal de Juiz de Fora Juiz de Fora Brazil; ^4^ Laboratório de Biotecnologia, Centro de Biociências e Biotecnologia Universidade Estadual do Norte Fluminense Darcy Ribeiro Rio de Janeiro Campos dos Goytacazes Brazil

**Keywords:** 5‐azacitidine, DNA hypomethylation, epigenetic regulation, microbiome, plant‐microorganism interaction

## Abstract

Microorganism and plant interactions are crucial for development and environmental adaptation. Plant growth promoting bacteria enhance agricultural productivity in a sustainable manner, while epigenetic modifications such as DNA methylation regulate gene expression and adaptive responses. The objective of this study is to determine how DNA hypomethylation influences early interactions between maize (*Zea mays*) and the endophytic diazotrophic bacterium *Herbaspirillum seropedicae*, particularly regarding plant growth, metabolism, and the root microbiome. Treatment with the hypomethylating agent 5‐azacytidine (5‐azaC) altered maize root morphology without affecting bacterial growth. Inoculation with *H. seropedicae* promoted plant growth and bacterial colonisation in root mucilage, with higher accumulation in 5‐azaC treated roots. Global methylation analysis showed that bacterial inoculation modulates cytosine methylation in a manner similar to 5‐azaC, suggesting a role in epigenetic regulation. Gene expression analysis of DNA methylation machinery confirmed that hypomethylation drives plant‐microbe interactions. Root microbiome profiling revealed that 5‐azaC disrupted microbial composition, which was partially restored by bacterial inoculation. Proteomic analysis identified 1,818 proteins and highlighted significant changes in metabolic pathways, especially carbon metabolism and the citric acid cycle. These findings demonstrate that DNA hypomethylation combined with bacterial interaction profoundly affects cellular and metabolic processes and provide insights for sustainable agricultural practices through epigenetic and microbial modulation.

## Summary statement

- DNA hypomethylation enhances *Herbaspirillum seropedicae* colonisation, reshapes the root microbiome and proteome, and reprogrammes maize metabolism, revealing DNA methylation as a central regulator of early plant–microbe interactions.

## 1 Introduction

The increasing demand for sustainable agriculture has stimulated the development of alternatives that reduce dependence on synthetic fertilisers and mitigate their ecological impact (Chen et al. 2018). Among these alternatives, bioinputs‐biological agents and natural compounds that improve crop productivity while preserving environmental integrity, have gained considerable attention. Within this group, plant growth‐promoting bacteria (PGPB) are particularly relevant due to their capacity to enhance plant growth, yield, and resilience through multiple mechanisms (Sharma 2016; Koskey et al. 2021; Liu‐Xu et al. 2024).

PGPB contribute to crop performance by facilitating nitrogen fixation, phosphate solubilisation, and the production of phytohormones that stimulate root and shoot development (Rodriguez et al. 2019; Timofeeva et al. 2023). These microorganisms establish complex molecular interactions with their hosts, triggering signalling cascades and transcriptional reprogramming that enhance nutrient uptake, stress tolerance, and defence responses (Chen et al. 2022; Kaleh 2024). Beyond these well‐known effects, PGPB can also improve the acquisition of essential micronutrients such as iron, zinc, and potassium, produce siderophores that increase iron availability, lower stress‐related ethylene levels through the activity of the enzyme 1‐aminocyclopropane‐1‐carboxylate deaminase activity, and activate systemic resistance pathways that strengthen plant immunity (Ferreira et al. 2019; Kumar et al. 2025; Maciel‐Rodríguez et al. 2025). As a result, PGPB have been widely associated with biofertilization, biostimulation, biocontrol, mitigation of abiotic stresses, and overall improvements in plant resilience across diverse crops (Kour et al. 2020; Sammauria et al. 2020; Tian et al. 2020).

Despite these advances, an important dimension remains underexplored: the epigenetic regulation of plant‐PGPB interactions. Epigenetic mechanisms govern gene expression without altering the DNA sequence, thereby enabling plants to fine‐tune developmental and adaptive responses to environmental cues. Among these mechanisms, DNA methylation is especially critical, directly influencing transcriptional activity and modulating responses to both beneficial and pathogenic microbes (Niederhuth and Schmitz 2014; Seymour and Becker 2017; Chen et al. 2022). In plants, methylation occurs in three sequence contexts‐CG, CHG, and CHH‐maintained by distinct enzymes such as MET1, CMT2/3, and DRM2, while active demethylation is catalysed by glycosylases including ROS1, DME, and related proteins (Law and Jacobsen 2010; Zhang et al. 2018; Thiebaut et al. 2019; Chen et al. 2022).

Emerging studies reveal that microbial associations can shape plant epigenomes. In Arabidopsis, PGPB have been shown to reconfigure DNA methylation landscapes, promoting root architectural changes and stress adaptation (Kawakatsu et al. 2016; Timofeeva et al. 2023). Similar effects have been described in other species, where PGPB‐induced epigenetic shifts correlate with enhanced nutrient assimilation, drought tolerance, biomass accumulation, and pathogen resistance (Chen et al. 2022). However, systematic investigations in staple crops remain limited.

Maize (*Zea mays L*.), one of the most important cereals worldwide, sustains global food security and provides both calories and essential nutrients for humans and livestock (Anami et al. 2009; Prasanna 2013; Tanumihardjo et al. 2020). As a model cereal with a complex but well‐annotated genome, maize offers unique opportunities to dissect how epigenetic regulation contributes to microbial interactions (Agostini et al. 2023; Wu and Fan 2025). Understanding these processes is fundamental for leveraging PGPB as bioinputs in crop improvement programmes.

In this study, we investigate how DNA hypomethylation shapes early interactions between maize roots and *Herbaspirillum seropedicae*, a well‐established endophytic diazotroph and PGPB in maize (Baldani 1986; Canellas and Olivares 2017; Rosman et al. 2024). Our aim is to determine how reducing DNA methylation influences root development, bacterial colonisation, and associated molecular responses. Using the methylation inhibitor 5‐azacytidine (5‐azaC), we assess changes in root architecture, microbiome composition, and proteomic profiles. Together, these analyses clarify the role of DNA hypomethylation in regulating plant–PGPB interactions.

## 2 Materials and Methods

### 2.1 Assessment of 5‐azaC Effect on Bacterial Growth

The bacterium used *was H. seropedicae strain* RAM10, a derivative of *H. seropedicae* strain ZA95 (Baldani 1986), which carries the *GFP* gene inserted via the transposon Tn5 in its chromosomal DNA. This strain was kindly provided by Dr. Rose Adele Monteiro (Department of Biochemistry and Molecular Biology, Federal University of Paraná, Brazil). The bacterium was growth in the presence of the DNA methylation inhibitor 5‐azaC was assessed using a 96‐well microplate assay. Bacterial cultures were initially grown in liquid DYGS medium (2 g of glucose, 2 g of malic acid, 1.5 g of bacteriological peptone, 2 g of yeast extract, 0.5 g of K₂HPO₄, and 0.5 g of MgSO₄·7H₂O, 1.5 g of glutamic acid, adjusted to pH 6.0.) at 30°C with agitation at 120 rpm until reaching an optical density at 600 nm (OD₆₀₀) of 1.0. For the assay, an inoculum corresponding to 10% of the final volume was added to each well, containing DYGS medium and different concentrations of 5‐azaC (2.5, 25, and 250 µM). A control without 5‐azaC was included to evaluate bacterial growth in the absence of the hypomethylating agent. Bacterial growth was monitored hourly by measuring the optical density (OD₆₀₀) over 16 h using a microplate spectrophotometer, with incubation at 30°C under continuous shaking at 120 rpm.

### 2.2 Plant Growth and Sterile Culture Conditions

Seeds of *Z. mays* (UENF 506‐11) were surface‐sterilised by immersion in 70% ethanol for 5 min, followed by treatment with 1.5% sodium hypochlorite for 30 min. Subsequently, seeds were rinsed six times with ultra‐pure water and soaked for 6 h. For germination, seeds were placed in Petri dishes (150 × 25 mm) containing 20 mL of water agar medium (5 g/L, semi‐solid), which had been previously sterilised by autoclaving at 120°C for 15 min. The plates were incubated in a growth chamber (B.O.D.) at 27°C under a 12/12 h light/dark photoperiod, with a light intensity of 100 µmol m⁻² s⁻¹ for 3 days. After this period, the germinated seedlings were transferred to sterile test tubes (25 × 150 mm) containing three glass beads (1.6 cm diameter) and 10 mL of half‐strength Murashige and Skoog (½ MS) medium, with the pH adjusted to 5.8. After 2 days, an additional 5 mL of ½ MS medium was added. The medium was autoclaved at 120°C for 15 min prior to use. Seedlings were maintained under the same growth conditions (27°C, 12/12 h photoperiod, 100 µmol m⁻² s⁻¹) for an additional 2 days, completing a total culture period of 7 days.

### 2.3 Application of the Hypomethylating Agent 5‐azaC

A stock solution of 5‐azaC was prepared at 100 mM, dissolved in dimethyl sulfoxide (DMSO). From this stock solution, a 10 mM solution of 5‐azaC was prepared in ultrapure water. The 5‐azaC solution was added to both the water agar and MS media at the desired concentrations. The treatment with 5‐azaC was applied in three stages: (i) during seed germination, where seeds were sown in Petri dishes containing water agarmedium (semi‐solid) with 0.25, 2.5 and 25 µM 5‐azaC; (ii) when transferring the seedlings to MS medium containing 0.25, 2.5 and 25 µM 5‐azaC; (iii) when the seedlings were inoculated with bacteria, the MS medium was also supplemented with 0.25, 2.5 and 25 µM 5‐azaC.

### 2.4 Plant Experimental Design and Bacterial Inoculation

The seedlings were distributed into the following experimental conditions: 5‐azaC treatment (A), where seedlings were treated with 5‐azaC during germination and growth, without bacterial inoculation; inoculation with *H. seropedicae* (B), where seedlings were inoculated with *H. seropedicae* without 5‐azaC treatment; 5‐azaC treatment and inoculation with *H. seropedicae* (AB), where seedlings were treated with 5‐azaC during germination and growth and later inoculated with *H. seropedicae*; and the Control (C), where maize seedlings were neither treated with 5‐azaC nor inoculated with *H. seropedicae*.

**Figure 1:**
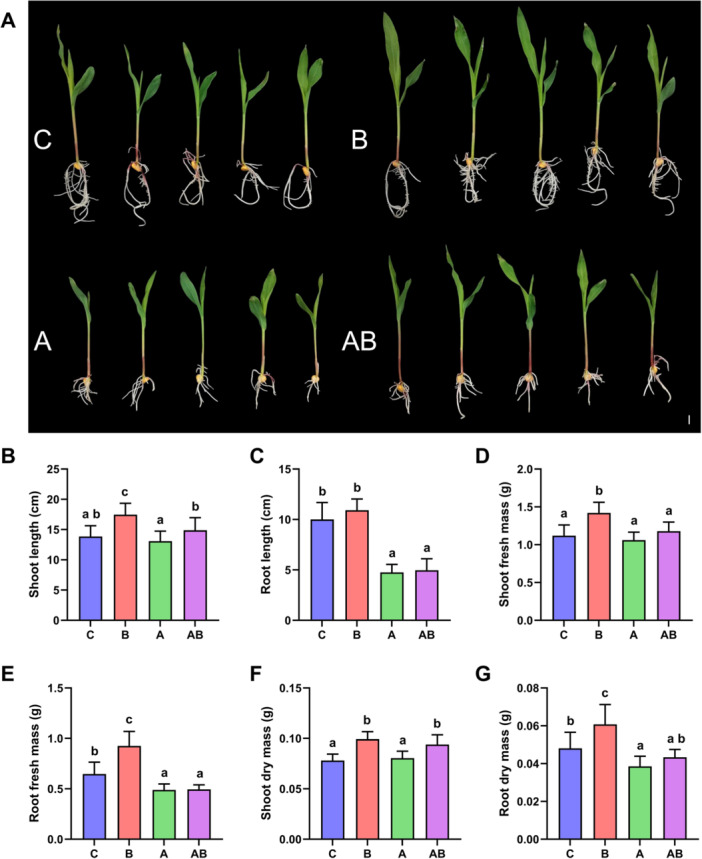
Effect of the methylation inhibitor (5‐azaC) on maize seedlings development at 7 DAI. Treatments included 2.5 µM 5‐azaC and inoculation with *H. seropedicae* for 48 HAI. (A) Image showing the effect of the compound on seedling growth (Scale bar = 1 cm). (B, D, F) Measurements of shoot length, fresh mass, and dry mass, respectively. (C, E, G) Measurements of root length, fresh mass, and dry mass, respectively. Letters (a, b, c, d) indicate significant differences between treatments, as determined by ANOVA followed by Tukey's test. DAI, days after imbibition; HAI, hours after inoculation. In the graphs, the labels C, B, A, and AB correspond to Control, Bacteria, 5‐azaC, and 5‐azaC + and Bacteria, respectively.

For inoculation, *H. seropedicae* cultures were centrifuged and the pellet resuspended in MS medium to a final concentration of 2 × 10⁶ bacteria/mL for application to 5‐day‐old seedlings. A schematic representation of the experimental design is shown in Figure 1S.

### 2.5 Biometric Analysis to Assess Plant Growth Promotion

The experiment was conducted using a completely randomised design (CRD), with twenty biological replicates for each treatment (C, B, A, AB). The seedlings were subjected to biometric evaluations, which included measurements of root and aerial segment length (cm), fresh weight, and dry weight. Fresh weight was determined immediately after the seedlings were collected. For dry weight determination, samples were incubated in a drying oven at 60°C for 24 h and subsequently reweighed.

### 2.6 Nucleic Acid Extraction and cDNA Synthesis

Genomic DNA from maize roots was extracted using the CTAB (Cetyltrimethylammonium bromide) method (Doyle and Doyle 1987; Chen and Ronald 1999). Total RNA was extracted using Trizol® (Invitrogen), following the manufacturer's instructions. Nucleic acid concentration and purity were assessed using a NanoDrop 2000/2000c spectrophotometer (Thermo Fisher Scientific). DNA and RNA integrity were verified by electrophoresis on a 1% agarose gel stained with ethidium bromide. cDNA was synthesised from 5 µg of RNA using the GoScript Reverse Kit (Promega), following the manufacturer's protocol.

### 2.7 RT‐qPCR Analysis of DNA Methylation Machinery and Bacterial Colonisation

The quantification of *H. seropedicae* followed the protocol described by (Da Silva et al. 2023), with modifications. The curve used for quantification is shown in Supporting Information Figure S2. For gene expression analysis, RT‐qPCR reactions were performed using SYBR Green PCR Master Mix (Applied Biosystems). Amplifications were performed using the Applied Biosystems QuantStudio Real‐Time PCR System, according to the manufacturer's recommendations, in 96‐well plates with a final volume of 10 µL. Each reaction contained 0.5 μL of each primer (forward and reverse), 5 μL of SYBR Green, 2 μL of cDNA, and 2.5 μL of ultrapure water. Primers were designed using the OligoAnalyzer tool from Integrated DNA Technologies (IDT) (Table S1). Relative gene expression was quantified using the 2^ − ΔΔCT method, as described by (Rao et al. 2013).

### 2.8 Microscopic Analysis of Root Colonisation and Morphology

For fluorescence microscopy, entire roots were placed on glass slides with sterile distilled water and observed under an ECLIPSE Ni (Nikon) fluorescence microscope, equipped with specific filters for GFP detection (BP 460‐490 nm; LP 510‐550 nm), and a Prime Vision FL digital photography system for image capture. Observations were performed on longitudinal sections of the pellucid zone of maize roots under conditions C, B, A and AB.

For Scanning Electron Microscopy (SEM) analysis, maize roots were cut into 1 cm long segments, including the root cap, elongation zone, and root hair zone, and immediately fixed in Karnovsky's solution (4% formaldehyde, 2.5% glutaraldehyde, 0.1 M sodium cacodylate buffer, pH 7.4). Samples were then washed with the same buffer (three times for 10 min), dehydrated in an ethanol series (15%, 30%, 50%, 70%, 90%, and 2 × 100% for 10 min each), and dried in a critical point drying device (Baltec CPD 030). The segments were mounted on aluminium stubs, sputter‐coated with ionised platinum (Bal‐tec SCD 050), and visualised using the (SEM) Zeiss EVO 40 SEM at 15 kV.

### 2.9 DNA Methylation Analysis

DNA extracted from the roots of different treatments (C, B, A, and AB) was enzymatically digested into nucleosides using the Nucleoside Digestion Mix (New England Biolabs). The digestion reaction was prepared by mixing 1 µg of DNA, 2 µL of 10× reaction buffer, and 1 µL of the digestion mix, with the final volume adjusted to 20 µL with ultrapure water. The mixture was incubated at 37°C for 24 h in a thermomixer, followed by incubation at 70°C for 10 min and centrifugation at 10,000 rpm for 10 min at 23°C. Eighteen microliters of the supernatant were transferred to a new tube and diluted 200‐fold prior to mass spectrometry analysis.

Chromatographic separation of nucleosides was performed using an Acquity UPLC I‐Class FTN system (Waters) equipped with an Acquity UPLC BEH C18 column (1.7 µm, 2.1 mm × 50 mm; Waters) maintained at 40°C. Ten microliters of the sample were injected and separated at a flow rate of 300 µL·min⁻¹ using a binary gradient composed of LC‐MS grade water with 0.01% formic acid (solvent A) and LC‐MS grade methanol with 0.01% formic acid (solvent B). The gradient programme was as follows: 0–5 min, 100% A to 95% A; 5–6 min, 95% A to 5% A; 6–7 min, held at 5% A; 7–8 min, returned to 100% A; 8–10 min, maintained at 100% A for column re‐equilibration.

Mass spectrometric detection was conducted on a Xevo TQ‐XS triple quadrupole mass spectrometer (Waters) equipped with a high‐performance Z‐spray electrospray ionisation (ESI) source operating in positive ion mode and multiple reaction monitoring (MRM). The cone voltage was set to 20 V, and the collision energy was set to 18 eV. Data acquisition and peak integration were performed using MassLynx (v4.2) and TargetLynx XS (Waters) software. The monitored MRM transitions (precursor/product, m/z) were: 5mdC (242/126), 5hmdC (258/142), dC (228/112), dG (268/152), dA (252/136), and dT (243/127). Quantification was achieved by comparison to standard curves generated from the 5‐Methylcytosine & 5‐Hydroxymethylcytosine DNA Standard Set (Zymo Research, D5405), which ranged from 0.1 to 15 ng.

To assess the cytosine methylation pattern in maize DNA, the isoschizomer enzymes HpaII and MspI were used. Genomic DNA was extracted and diluted in ultrapure water to a final concentration of 25 ng/μL. Approximately 250 ng of DNA from each sample was digested with 5 U of HpaII and MspI (Promega) in the presence of 1× reaction buffer and ultrapure water, in a final volume of 50 μL per sample, at 37°C for 2 h. Subsequently, the samples were amplified by PCR using ISSR markers (Table S2). PCR reactions were performed in a final volume of 25 μL containing 5 μL of GoTaq Flexi buffer (Promega), 0.5 μM primer, 0.15 mM dNTPs, 1U of Taq DNA polymerase (Promega), 3 mM MgCl₂, and 15 ng of DNA. The amplification programme consisted of an initial denaturation step at 94°C for 4 min, followed by 45 cycles of denaturation at 94°C for 45 s, annealing at a temperature optimised for each primer (Table S2), extension at 72°C for 2min, and a final extension step at 72°C for 7 min.

PCR products were separated by electrophoresis in 1× TBE buffer and 2% agarose gels. The gels were stained with SYBR Safe DNA Gel Stain (Sigma) and visualised using a UV transilluminator. Fragment sizes were estimated using a 100 bp molecular weight marker (Amresco). For quantification of global DNA methylation, PCR amplification patterns were recorded as a binary matrix, where bands of similar size were classified as 1 (band present) or 0 (band absent) (Table S3).

### 2.10 Quantification of Fluorescent Inoculum Colony Forming Units (CFUS)

After 7 days of growth under the different conditions (C, B, A, AB), the plant roots were collected. For bacterial extraction, one gram of root was macerated in sterile 0.85% saline solution and the resulting suspension was then subjected to serial dilutions.

The quantification of colony‐forming units (CFUs) was performed using the Drop Plate technique (Herigstad et al. 2001). The culture medium used was DYGS solid, prepared with the following composition (per 1 L of medium): 2 g of glucose, 2 g of malic acid, 1.5 g of bacteriological peptone, 2 g of yeast extract, 0.5 g of K₂HPO₄, 0.5 g of MgSO₄·7H₂O, 1.5 g of glutamic acid, and 15 g of agar, adjusted to pH 6.0.

Serial dilutions were applied to the Petri dishes, which were then incubated at 30°C for 20 h in a thermostat. The quantification of CFUs was performed using a fluorescence microscope equipped with filters specific for GFP detection (excitation BP 460–490 nm; emission LP 510–550 nm). Only the fluorescent colonies were counted, ensuring that the quantification was specific to the bacterial inoculum.

### 2.11 Metataxonomic Analysis

The DNA extracted from the roots of the different conditions (C, B, A, and AB) was sent to the company Genone for sequencing of the 16S rRNA gene using the Illumina MiSeq platform, with three replicates per condition. The quality and quantity of the extracted DNA were examined using electrophoresis on a 1.8% agarose gel, and DNA concentration and purity were determined using a NanoDrop 2000 UV‐Vis Spectrophotometer (Thermo Scientific, Wilmington, USA). The hypervariable region V3‐V4 of the bacterial 16S rRNA gene was amplified using the primer pairs 338 F (5'‐ ACTCCTACGGGAGGCAGCA‐3') and 806 R (5'‐ GGACTACHVGGGTWTCTAAT‐3'). Both the forward and reverse 16S primers were tailed with sample‐specific Illumina index sequences to allow for deep sequencing. The PCR was performed in a total reaction volume of 10 μL: DNA template 5–50 ng, forward primer (10 μM) 0.3 μL, reverse primer (10 μM) 0.3 μL, KOD FX Neo Buffer 5 μl, dNTP (2 mM each) 2 μL, KOD FX Neo 0.2 μL, and finally ddH2O up to 20 μL, after initial denaturation at 95°C for 5 min, followed by 20 cycles of denaturation at 95°C for 30 s, annealing at 50°C for 30 s, and extension at 72°C for 40 s, and a final step at 72°C for 7 min. The amplified products were purified using the Omega DNA purification kit (Omega Inc., Norcross, GA, USA) and quantified with the Qsep‐400 (BiOptic Inc., New Taipei City, Taiwan, ROC). The amplicon library was paired‐end sequenced (2 × 250) on an Illumina NovaSeq. 6000.

Demultiplexed 16S rRNA gene sequences were processed using Qiime2 (Bolyen et al. 2019). Primer sequences were trimmed with cutadapt (Martin 2011), and denoising, chimera removal, and ASV inference were performed using DADA2 (Callahan et al. 2016). Taxonomic classification was based on the Greengenes 2 database (McDonald et al. 2023).

The resulting. QZA files were imported into R (R Core Team 2024) using the qiime2R package (Jordan and Bisanz, 2018). Alpha diversity (Shannon and Chao1) and beta diversity (Bray–Curtis followed by PCoA) were calculated using the phyloseq package (McMurdie and Holmes, 2013). Genus‐level taxonomy and Venn diagrams were generated using microeco (Liu et al. 2021), which also supported functional prediction based on FAPROTAX (Louca et al. 2016).

ASVs with fewer than 20 reads in < 10% of samples were removed prior to bipartite network construction. The specificity and fidelity of taxa among treatments were determined using the multipatt() function from indicspecies (Cáceres and Legendre, 2009), with 9,999 permutations. Adjusted p‐values were obtained with the qvalue package, and significant associations (q < 0.05) were visualised using ggraph. All other plots were produced with ggplot2 (Wickham 2016).

### 2.12 Label‐Free Proteomic Analysis

Roots from maize seedlings subjected to treatments C, B, A, and AB after 7 days of growth were collected for proteomic analysis. Three biological replicates per treatment (300 mg fresh mass) were ground in liquid nitrogen and resuspended in 1 mL of extraction buffer (10% TCA/acetone, 20 mM DTT). Samples were vortexed for 30 min at 4°C, incubated at −20°C for 1 h for precipitation, and centrifuged (16,000 g, 30 min, 4°C). The pellet was discarded, and the protein concentration in the supernatant was determined using the Bradford assay (Bio‐Rad) with BGG as the standard.

Proteins were solubilized in a 7 M urea and 2 M thiourea solution and digested with trypsin using Microcon‐30 kDa filter units (Millipore), following the FASP protocol (Wiśniewski et al. 2009) with modifications. Peptides were quantified using a NanoDrop 2000c spectrophotometer (Thermo Fisher Scientific), and 1 μg was injected into a nanoAcquity UPLC system coupled to a SYNAPT G2‐Si Q‐TOF mass spectrometer (Waters, Manchester, UK), as described by Botini et al. (2021).

Proteomic analysis was performed using ProteinLynx Global SERVER (PLGS) v.3.02 (Waters), and label‐free quantification was conducted with ISOQuant v.1.7 (Distler et al. 2014). Differential protein abundance was assessed by comparing the following ratios: B/C, A/C, AB/B, and AB/A. The ratios AB/B and AB/A were specifically used to infer the synergistic effects of the combined treatment. Significance was determined using a two‐tailed Student's *t*‐test, considering proteins as significantly up‐ or down‐accumulated if *p* ≤ 0.05 and log₂ fold‐change ≥ 0.5 or ≤ −0.5. Functional annotation of differentially expressed proteins was performed in ShinyGO (https://bioinformatics.sdstate.edu/go/) using KEGG pathway analysis to identify key metabolic pathways. The proteomics data have been deposit in the PRIDE repository under accession number PXD077098. The list of differentially accumulated proteins is available as supporting information.

## 3 Results

### 3.1 Effect of 5‐azaC on Bacterial and Maize Seedling Growth

The bacterium *H. seropedicae* was selected because it is a well‐established endophytic and diazotrophic PGPB in maize. The growth dynamics, illustrated in Figure S3, indicate that 5‐azaC does not alter bacterial proliferation within the tested concentration range. Growth was assessed under treatments of 2.5 μM, 25 μM, and 250 μM of 5‐azaC, and compared to untreated controls. These results demonstrate that 5‐azaC does not compromise bacterial growth, supporting its suitability for studies focused on epigenetic interactions without unintended effects on bacterial viability.

The screening of 5‐azaC concentrations revealed a dose‐dependent effect on maize seedling development. At 25 µM (Figure S4), a severe impairment in root development was observed, while bacterial viability remained unaffected in the hypomethylated treatments (A and AB). At 2.5 µM (Figure 1), the hypomethylation effect was less harmful but still evident. Under this condition, significant increases in shoot length and dry mass were detected in treatments A and AB. In contrast, the lowest concentration tested (0.25 µM, Figure S5) resulted in minor phenotypic effects, with no substantial changes in plant growth or evidence of enhanced plant‐bacteria interaction. Comparing the control (C) and bacteria‐only (B) treatments revealed that treatment B led to significantly increased shoot length, fresh mass, and dry mass, suggesting a positive effect of the bacteria on seedling growth. Based on these observations, concentration of 2.5 µM was selected for further experiments. These results indicate that 5‐azaC‐induced DNA hypomethylation, in combination with *H. seropedicae* inoculation, is associated with enhanced plant growth and increased bacterial colonisation, suggesting a positive early interaction.

### 3.2 DNA Methylation Modulated by Bacterial Inoculation and 5‐azac

**Figure 2:**
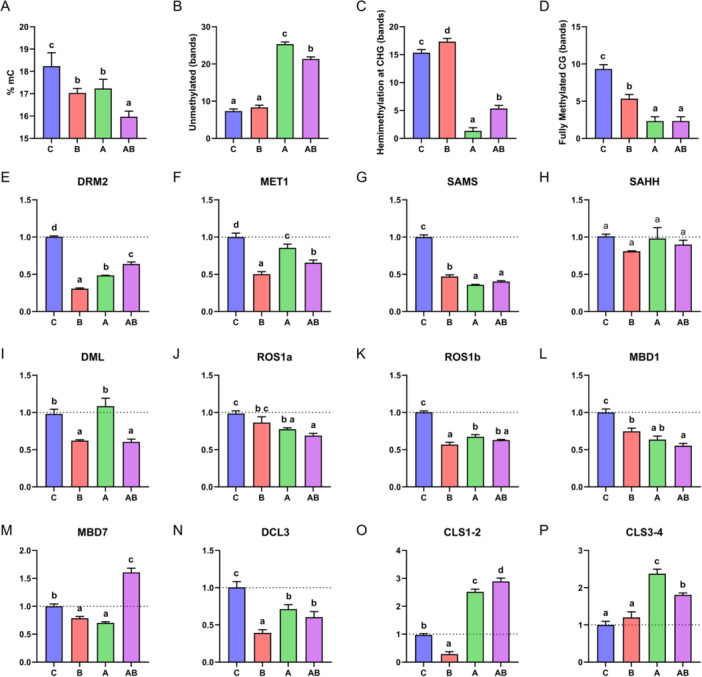
DNA methylation levels and expression of genes related to DNA methylation in maize seedling roots. Seedlings were treated with the methylation inhibitor 5‐azaC at 2.5 µM for 7 DAI and inoculated with *H. seropedicae* for 48 HAI. Global cytosine methylation (A), unmethylated cytosines (B), hemimethylation at CHG sites (C), and fully methylated CG sites (D). (E–P) Expression of genes related to DNA methylation and epigenetic modulation. Letters (a, b, c, d) in panels A–D indicate significant differences between treatments based on the Mann–Whitney test, while in panels E–P letters indicate differences based on Tukey's test. Treatments are represented as follows: C (Control), B (Bacteria), A (5‐azaC), and AB (5‐azaC + Bacteria). Data are presented as mean percentages with standard error. [Color figure can be viewed at wileyonlinelibrary.com]

To understand the effects of the hypomethylating agent 5‐azaC on DNA methylation in maize roots, we first evaluated the methylation profiles across treatments (Figure 2). As expected, a significant reduction in DNA methylation was observed in plants treated with 5‐azaC, consistent with its role as an inhibitor of DNA methylation. Interestingly, the bacterial inoculation (*H. seropedicae*) also caused a noticeable reduction in global DNA methylation.

**Figure 3:**
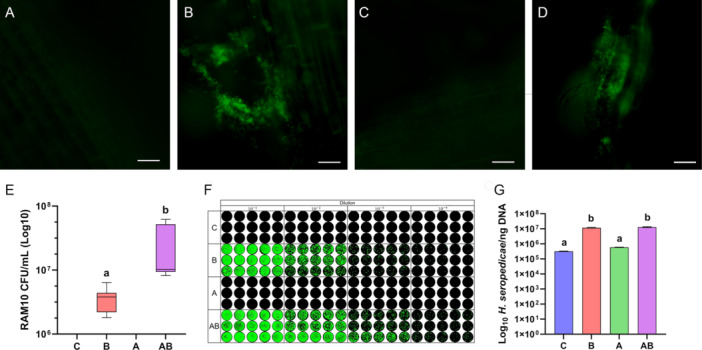
Fluorescence microscopy and quantification of H. seropedicae in maize seedling roots. Seedlings were treated with the DNA methylation inhibitor 5‐azaC (2.5 µM) for 7 DAI and inoculated with *H. seropedicae* expressing the fluorescent marker GFP (RAM10) for 48 HAI (A‐D) or for 24 HAI for bacterial quantification (E‐G). Shows longitudinal sections of the pellucid zone: (A) Control, (B) Bacteria‐only treatment, (C) 5‐azaC‐only, and (D) 5‐azaC + Bacteria treatment. Scale bar: 40 µm. Bacterial quantification: (E) colony‐forming units (CFU/mL), (F) visualisation of CFUs and their serial dilutions, and (G) total bacterial quantification of *H. seropedicae* by RT‐PCR. Treatments are represented as follows: C (Control), B (Bacteria), A (5‐azaC), and AB (5‐azaC + Bacteria). Different letters (a, b, c, d) indicate significant differences among treatments based on ANOVA followed by Tukey's test. [Color figure can be viewed at wileyonlinelibrary.com]

In plants treated with both 5‐azaC and bacteria (AB), cytosine methylation levels were significantly lower than in plants treated with 5‐azaC alone (Figure 2A). The proportion of unmethylated cytosines (Figure 2B) was greater in 5‐azaC treatments (A and AB). The hemimethylated CHG sites (Figure 2C) exhibited a greater reduction in response to 5‐azaC treatments (A and AB), whereas bacterial treatment (B) increased. The bacterial treatment (B) also affected the fully methylated CG sites (Figure 3D), showing a decrease similar to that observed in 5‐azaC treatments (A and AB). Interestingly, an intermediate methylation profile was identified in treatment B compared to treatments A and AB (Figure 2), characterised by a slight reduction in the proportion of unmethylated cytosines and a modest increase in CHG hemimethylation relative to treatment A.

We next evaluated the impact of each treatment on the expression of DNA methylation‐related genes, including writers, erasers, and readers. Gene expression analysis revealed that *H. seropedicae* inoculation (B), 5‐azaC treatment (A), and their combination (AB) differentially modulated epigenetic regulators in maize roots. Among the writers, DRM2 and MET1 showed a significant reduction in all treatments, with a markedly downregulated expression in B compared to the control. In the methyl cycle, S‐ADENOSYLMETHIONINE SYNTHETASE (SAMS) was consistently downregulated in B, A, and AB, while S‐ADENOSYL‐l‐HOMOCYSTEINE HYDROLASE (SAHH) expression remained stable. Among the erasers, DML was downregulated in B and AB but remained unchanged in A. Similarly, ROS1a followed the same general trend with less pronounced variation, showing its highest levels in AB, while ROS1b expression was reduced across all treatments (B, A, and AB).

Regarding the readers, MBD1 expression decreased across all treatments, whereas MBD7 was induced explicitly in AB and repressed in both B and A. For the RNA‐directed pathways, DICER‐LIKE 3 (DCL3) was suppressed in all treatments, with B showing the lowest levels. CLASSY (CLS1‐2) decreased in B but increased in A and AB, whereas CLS3‐4 was also upregulated in A and AB. These collective changes, where *H. seropedicae* inoculation mirrors the 5‐azaC‐induced hypomethylation in CG context and distinctively modulates CHG methylation, strongly imply that the bacterium actively engages with and fine‐tunes the host's epigenetic machinery. This suggests that the plant's epigenetic landscape is a critical regulatory layer governing the early stages of the beneficial plant‐microbe interaction.

### 3.3 Colonisation of Maize Roots by *H. seropedicae* and 5‐azaC Treatment

Fluorescence microscopy confirmed the colonisation of maize roots by *H. seropedicae* strain RAM10 (GFP‐tagged). Fluorescent signals were detected near root hairs in B (bacteria) and AB (5‐azaC + bacteria) treatments, whereas no fluorescence was observed in C (control) or A (5‐azaC alone) (Figure 3A–D). The root hair zone was analyzed because it represents the primary site of early bacterial colonisation. Notably, the fluorescence signal in panel D (AB) was stronger than in panel B (B), directly supporting the subsequent CFU data. Quantification of bacterial colonisation by CFU counts revealed significantly higher bacterial loads in B and AB compared to C and A (Figure 3E,F). Notably, the AB treatment exhibited the highest colonisation levels. RT‐PCR quantification corroborated these findings, showing elevated *H. seropedicae* abundance in AB roots (Figure 3G).

**Figure 4:**
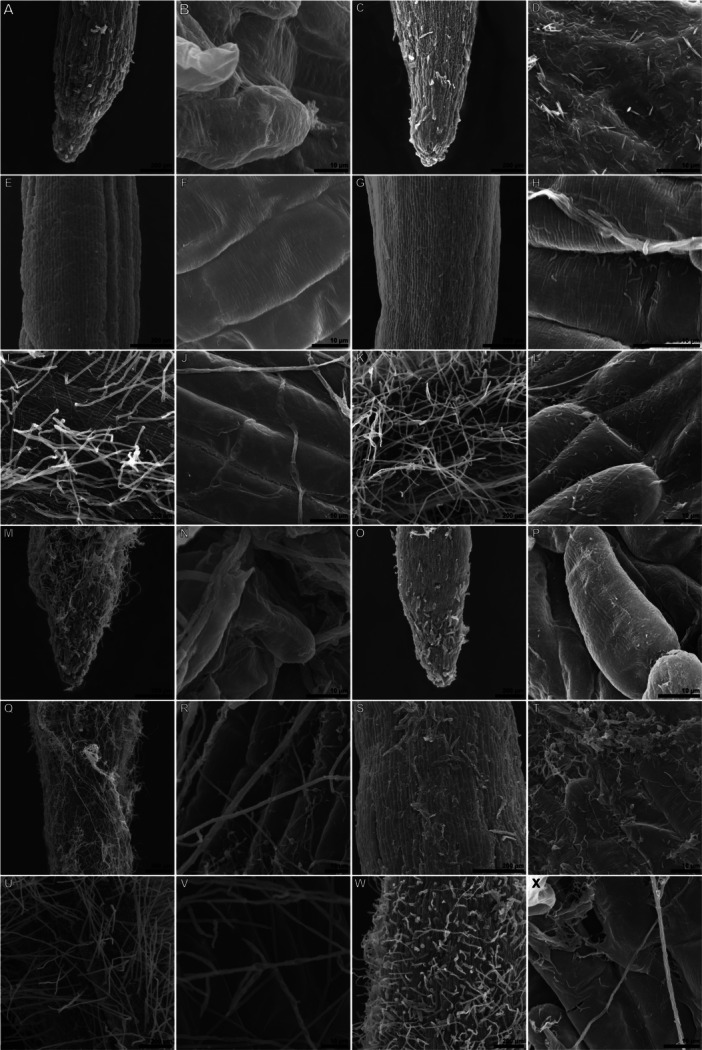
Scanning electron microscopy of maize seedling roots treated with the DNA methylation inhibitor 5‐azaC (2.5 µM) for 7 DAI and inoculated with *H. seropedicae* for 48 HAI. The analysis highlights three root zones: the root cap (images A–D and M‐P), the elongation zone (images E–H and Q–T), and the root hair zone (images I–L and U–X). Treatments are as follows: Control (C) includes images A, B, E, F, I, J; Bacteria (B) includes images C, D, G, H, K, L; 5‐azaC (A) includes images M, N, Q, R, U, V; and 5‐azaC + Bacteria (AB) includes images O, P, S, T, W, X.

SEM analysis showed no microbial structures in control roots (C), confirming effective surface sterilisation (Figure 4). In B‐treated roots, bacteria were distributed throughout the root cap, elongation zone, and pellucid zone. In A‐treated roots, dense fungal colonisation was observed in the elongation zone. In AB‐treated roots, bacteria were primarily localised in the elongation and pellucid zones, with no fungal presence detected. Taken together, these results demonstrate that the hypomethylation induced by 5‐azaC treatment significantly enhances the colonisation efficiency of the inoculated H. seropedicae on maize roots.

### 3.4 Root Microbiome Dynamics in Maize Under 5‐azaC Treatment and Bacterial Inoculation

**Figure 5:**
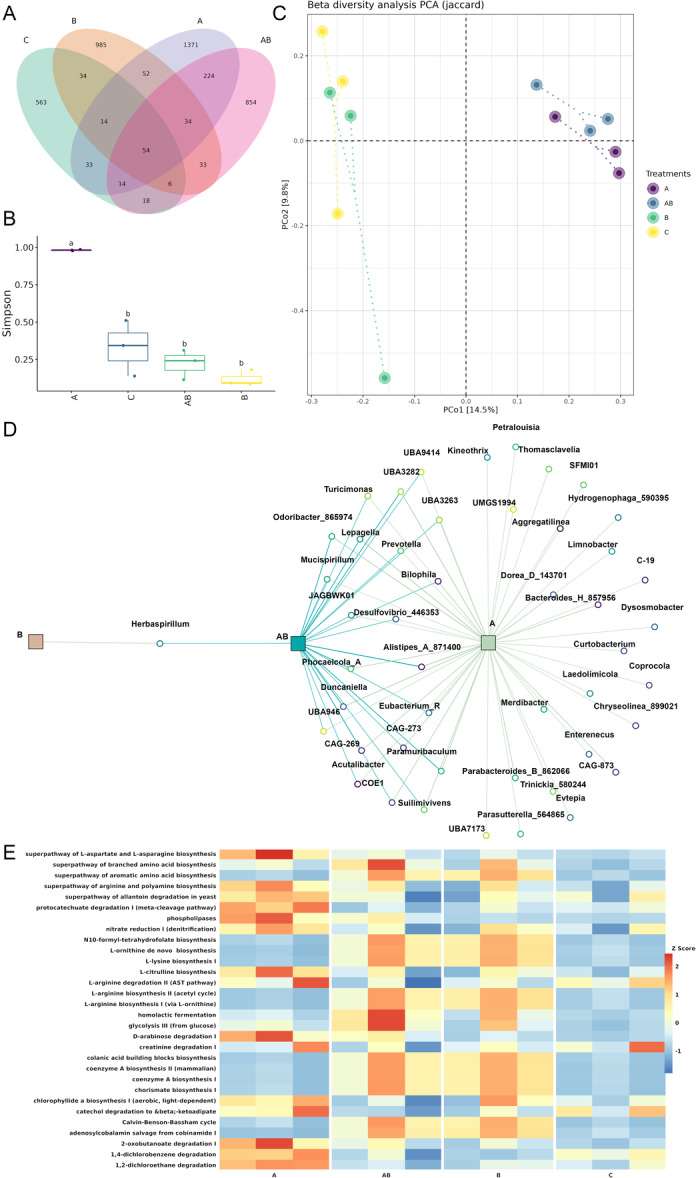
Microbial community structure and functional predictions in maize seedling roots under DNA methylation inhibition and bacterial inoculation. Seedlings were treated with the DNA methylation inhibitor 5‐azaC (2.5 µM) and inoculated with *H. seropedicae*. Variance analysis of the microbial community: (A) Venn diagram of OTU/ASV analysis, (B) boxplot of alpha diversity, and (C) PCA plot of beta diversity. Network of significant microbial associations (D), where nodes represent bacterial genera and edges indicate significant associations (*p* < 0.05). Heatmap of functional predictions of microbial associations (E). Treatments are represented as follows: C (Control), B (Bacteria), A (5‐azaC), and AB (5‐azaC + Bacteria). [Color figure can be viewed at wileyonlinelibrary.com]

The Venn diagram (Figure 5A) illustrates the distribution of unique and shared OTUs/ASVs among treatments. Groups C, B, and AB showed comparable profiles, whereas the 5‐azaC treatment (A) displayed a noticeably higher number of unique microbial taxa. This pattern indicates that DNA hypomethylation strongly alters the composition of the root microbiome.

Alpha diversity analysis (Figure 5B) revealed significant differences, with group A exhibiting a marked increase in diversity, whereas groups B, C, and AB showed no significant variations. After 48 h, bacterial interaction in AB did significantly alter diversity, indicating a stabilising effect. This interpretation is supported by the observation that the high diversity induced by 5‐azaC alone (Group A) was mitigated in the presence of *H. seropedicae* (Group AB), returning the alpha diversity to a level statistically similar to the control (C) and bacteria‐only (B) groups.

Beta diversity analysis using PCA (Figure 5C) revealed distinct microbial profiles among treatments. Groups C and B clustered together, as did A and AB, suggesting similarities in microbial composition within these pairs. The bacterial treatment alone did not form a distinct cluster, reinforcing the impact of DNA hypomethylation on microbiome structure.

The taxonomic distribution of bacteria in maize seedling roots (Figure S6) illustrates how treatments influenced the microbiome composition. At the genus level, Herbaspirillum showed notable enrichment in treatments C, B, and AB, and a reduction in A. The high relative abundance in the control group (C) suggests the presence of native *Herbaspirillum* strains, likely originating from the seed microbiome or the growth medium. The reduction of native *Herbaspirillum* in A indicates that the hypomethylation induced by 5‐azaC creates a root environment that is generally unfavourable for the colonisation of these native strains. Conversely, the successful enrichment in AB suggests that this negative effect can be overcome by the high‐dose, specific inoculant, as previously demonstrated in Figures 3 and 4. The most precise quantification of *Herbaspirillum* was presented in Figure 3, using RT‐PCR.

Bacteria from the genera *Duncanella, Alistipes*, and *Prevotella* were more abundant in treatment A and also present in AB, albeit at reduced levels—possibly due to the competitive effects of *H. seropedicae* inoculation. *Lepagella and Curtobacterium* were significantly enriched in treatment A. On the other hand, *Sphingomonas* and *Acinetobacter* were more abundant in treatments A and C, indicating that modulation was driven by plant status, regardless of whether DNA methylation was altered. Notably, treatment A exhibited the highest relative abundance and diversity of bacterial taxa, reinforcing the idea that DNA hypomethylation significantly shapes and modulates the root microbiome composition (Supporting Information Figure S6).

When analyzing the network of significant associations (Figure 5D), a clear connection is observed between treatments B and AB, involving only the genus *Herbaspirillum* of the inoculant species. In contrast, a much broader set of connections was identified between treatments A and AB, including genera such as *Mucispirillum, Odoribacter, Turicimonas, Lepagella, Prevotella, Bilophila, Desulfovibrio, Alistipes, Phocaeicola, Duncaniella, Eubacterium, Paramuribaculum, Acutalibacter*, and *Suilimivivens*. These findings suggest a more robust network of microbial interactions among the hypomethylated treatments. Notably, treatment C did not show any significant associations in the analysis and, therefore, is not included in the network graph.

Functional predictions based on microbial community composition revealed apparent differences in metabolic profiles across treatments (Figure 5E). Seedlings exposed to 5‐azacytidine (A) showed a pronounced enrichment of amino acid biosynthetic processes and organic compound degradation pathways, reflected by higher Z‐scores compared to the control. In contrast, the combined treatment with 5‐azacytidine and bacteria (AB) was associated with an increased representation of glycolysis, fermentation, and energy metabolism pathways, indicating a stronger reprogramming of the microbial functional potential driven by the joint effects of epigenetic modulation and bacterial inoculation.

The bacteria‐only treatment (B) exhibited intermediate functional shifts, characterised by a moderate induction of amino acid biosynthesis and Calvin‐Benson‐Bassham cycle pathways, albeit at a lower intensity than observed in the AB group. Meanwhile, the control (C) exhibited the most stable profile, characterised by lower functional variation and reduced enrichment across metabolic pathways.

Together, these findings demonstrate that both epigenetic modulation by 5‐azacytidine and inoculation with *H. seropedicae* significantly reshape the functional potential of the root‐associated microbiota, with the most pronounced effects arising from their combined application.

### 3.5 Proteomic Modulations Induced by DNA Demethylation and Bacterial Interaction

To investigate how changes in DNA methylation, gene expression, and root microbiome composition shape the maize root proteome, we performed a label‐free quantitative proteomic analysis across the four treatments (C, B, A, and AB). In total, 1,818 proteins were identified, and pairwise comparisons (B/C, A/C, AB/A, AB/B) revealed distinct sets of differentially accumulated proteins (DAPs). In Supporting Information Figure S7, up‐ and down‐accumulated proteins are indicated by blue and red arrows, respectively, while proteins unique to a single treatment are reported separately.

The B/C comparison revealed a relatively balanced response, with 23 proteins upregulated and 30 down‐regulated, in addition to several proteins detected exclusively in either condition. In contrast, the A/C comparison revealed a more substantial shift in abundance patterns, with 54 proteins accumulating at higher levels and 66 at lower levels, underscoring the pronounced effect of DNA methylation inhibition on the proteome.

In the AB/A comparison, the addition of bacteria to 5‐azaC‐treated roots resulted in 32 proteins showing increased and 37 showing decreased accumulation, alongside condition‐specific proteins. The AB/B comparison revealed the most pronounced alterations, with 96 proteins upregulated and 59 downregulated, highlighting the synergistic impact of DNA demethylation and bacterial colonisation on root proteome remodelling.

**Figure 6:**
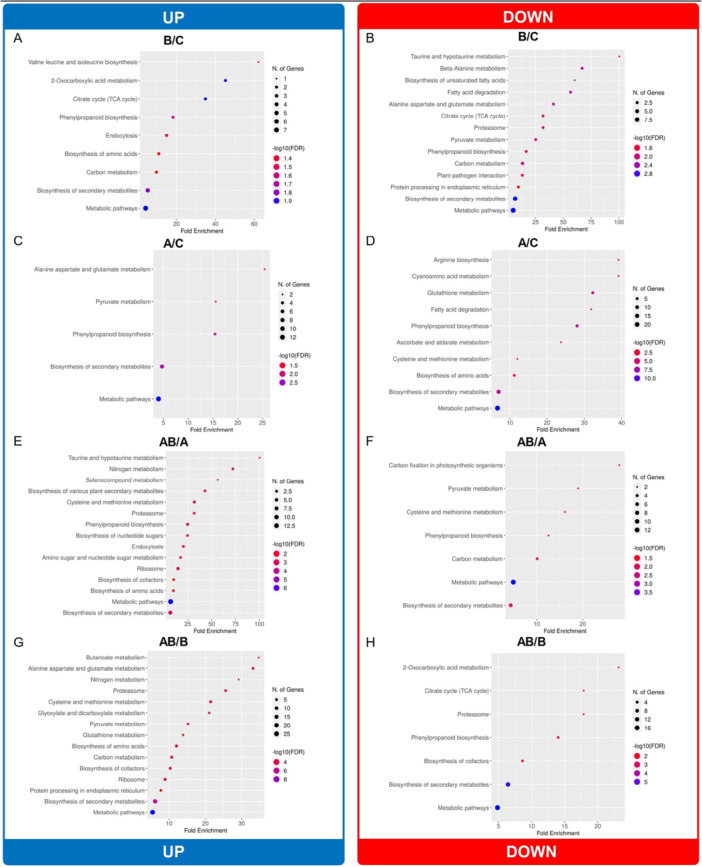
Gene ontology enrichment of differentially accumulated proteins in maize seedling roots is treated with the DNA methylation inhibitor 5‐azaC (2.5 µM) for 7 DAI and inoculated with *H. seropedicae* for 48 HAI. Comparisons: B/C (A, B), A/C (C, D), AB/A (E, F), AB/B (G, H). KEGG functional enrichment of differentially accumulated proteins, with bubble maps showing upregulated (A, C, E, G) and down‐regulated (B, D, F, H) pathways. [Color figure can be viewed at wileyonlinelibrary.com]

To determine the functional annotation of differentially accumulated proteins, a KEGG functional enrichment analysis was conducted using ShinyGO. Figure 6 illustrates the differentially accumulated proteins, along with their respective functional annotations, for both upregulated and downregulated proteins in the B/C, A/C, AB/A, and AB/B comparisons.

Overall, the treatments induced a complex and multifaceted regulation of various metabolic and signalling pathways, reflecting the physiological adaptations of the plants to the treatments. Significant modulation was observed in pathways related to primary and secondary metabolism, as well as in processes crucial for plant‐microorganism interaction. Among the most prominent pathways, carbohydrate metabolism and the citric acid cycle (TCA) demonstrated differential regulation across multiple comparisons. Specifically, the citric acid cycle (TCA) exhibited dual regulation in the B/C comparison, with both upregulated (Figure 6A) and down‐regulated (Figure 6B) proteins, indicating a complex reconfiguration of metabolic flux. In the AB/B comparison, this pathway was exclusively down‐regulated (Figure 6H), suggesting a decrease in oxidative activity when 5‐azaC was applied to the plants. Carbon metabolism, in turn, was differentially expressed in various comparisons, except for the A/C and AB/A comparisons. Notably, carbon fixation in photosynthetic organisms was downregulated only in the AB/A comparison (Figure 6F), which may indicate an alteration in photosynthetic efficiency following bacterial inoculation with 5‐azaC. Furthermore, the 2‐oxocarboxylic acid metabolism pathway was upregulated in the B/C comparison (Figure 6A) and downregulated in the AB/B comparison (Figure 6H), suggesting that the interaction between the plant and the bacterium without hypomethylation may specifically modulate this pathway.

The phenylpropanoid biosynthesis pathway showed regulation in almost all comparisons. The only exception was the AB/B comparison, where no positive regulation was observed (Figure 6G), indicating a more restricted response or a lower demand for phenylpropanoid‐derived compounds under this condition. Another relevant finding was the positive regulation of proteins related to the nitrogen fixation pathway, observed in the AB/A (Figure 6E) and AB/B (Figure 6G) comparisons, which may indicate increased nitrogen assimilation or a more efficient symbiotic interaction when 5‐azaC was applied. In contrast, the negative regulation of a plant‐pathogen interaction‐related protein in the B/C comparison (Figure 6B) suggests that the bacterial interaction has an impact on the plant's response, possibly modulating susceptibility or resistance.

## 4 Metabolic Pathways Altered by 5‐azaC Treatment and Bacterial Inoculation

**Figure 7:**
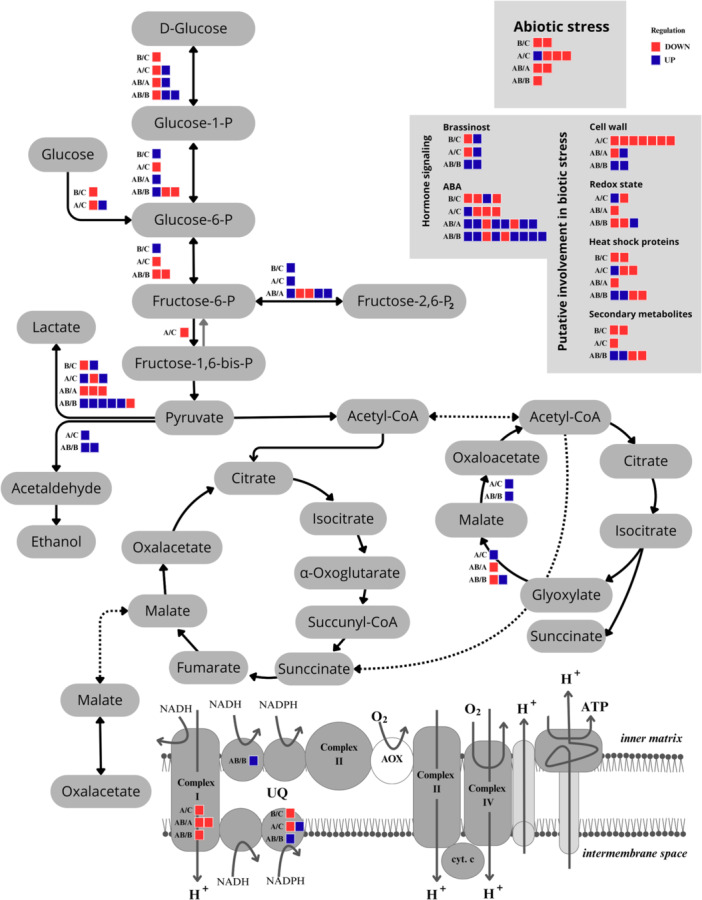
Functional mapping of proteomic changes in maize seedling roots under different treatments. Proteins identified in proteomic analysis were mapped onto central metabolic pathways, including glycolysis, the tricarboxylic acid (TCA) cycle, mitochondrial electron transport chain, and stress‐related processes. Coloured squares represent differential protein abundance across the following treatments: B/C (Bacteria vs. Control), A/C (5‐azacytidine vs. Control), AB/A (5‐azacytidine + Bacteria vs. 5‐azacytidine), and AB/B (5‐azacytidine + Bacteria vs. Bacteria). Blue indicates upregulation, while red indicates downregulation, relative to the comparison group. Functional clusters related to abiotic stress, biotic stress responses, and hormone signalling are highlighted in grey boxes. [Color figure can be viewed at wileyonlinelibrary.com]

Proteomic analysis revealed substantial remodelling of primary and secondary metabolism in maize under different treatments (Figure 7 and Table S4). In carbohydrate and energy metabolism, treatment B triggered increased carbon flux through glycolysis and optimised tricarboxylic acid (TCA) cycle activity, as indicated by the upregulation of DIHYDROXYACID DEHYDRATASE and ACONITASE HYDRATASE and downregulation of PYRUVATE DEHYDROGENASE KINASE and CYTOSOLIC NAD‐DEPENDENT MALATE DEHYDROGENASE. The treatment A reconfigured glycolysis and biosynthetic precursor supply, with upregulation of PLASTIDIAL PYRUVATE KINASE and repression of 2‐ISOPROPYLMALATE SYNTHASE. The treatment AB enhanced energy production and stress adaptation by predominantly upregulating glycolytic enzymes.

In amino acid metabolism, the metabolism of GABA was prominently affected. Treatment B reduced GABA shunt activity through downregulation of GLUTAMATE DECARBOXYLASE. In contrast, treatment A induced GLUTAMATE DECARBOXYLASE and GABA PYRUVATE TRANSAMINASE, while repressing METHYL‐TETRAHYDROFOLATE‐DEPENDENT METHIONINE SYNTHASE, thereby disrupting methionine and S‐adenosylmethionine metabolism. The treatment AB counterbalanced these opposing effects, resulting in a more stable metabolic state that supports both plant growth and defence when compared to the control C.

Secondary metabolism was modulated primarily through PHENYLALANINE AMMONIA LYASE, which was downregulated in B plants, favouring bacterial colonisation, but strongly induced in the AB treatment, reflecting activation of plant defence pathways. Lipid metabolism was intensified under A and AB treatments, with upregulation of TRIACYLGLYCEROL LIPASE‐LIKE 1 and ACYL‐CoA OXIDASE, suggesting membrane remodelling and enhanced energy production.

Nucleotide and protein biosynthesis were mainly affected by treatment A, as indicated by the upregulation of URIDINE 5'‐MONOPHOSPHATE SYNTHASE, RIBONUCLEOSIDE‐DIPHOSPHATE REDUCTASE, and the translation initiation factor EUKARYOTIC INITIATION FACTOR 4A‐2. Under treatment A, ribosomal proteins showed contrasting regulation, with induction of 40S RIBOSOMAL PROTEIN S8‐1 and repression of 40S RIBOSOMAL PROTEIN S15A‐1, 40S RIBOSOMAL PROTEIN S12, and 40S RIBOSOMAL PROTEIN S19‐3, potentially linked to abscisic acid signalling. In the AB treatment, the translation machinery was reinforced, supporting growth and adaptation.

Antioxidant defences were broadly suppressed by A treatment, including GLUTATHIONE PEROXIDASE, GLUTATHIONE S‐TRANSFERASE IV, ASCORBATE PEROXIDASE, PEPTIDE METHIONINE SULFOXIDE REDUCTASE, and 4‐HYDROXYPHENYLPYRUVATE DIOXYGENASE, indicating reduced oxidative stress tolerance. Redox imbalance was partially alleviated by bacterial inoculation in AB treatment.

Finally, cytoskeletal integrity was affected under A treatment, as evidenced by opposite regulation of ACTIN FILAMENT PROTEIN and repression of ACTIN DEPOLYMERIZING FACTOR, with AB treatment partially mitigating these effects. Ribosomal components associated with ABA signalling showed mixed regulation, including up‐regulation of 40S RIBOSOMAL PROTEIN S8‐1 and down‐regulation of 40S RIBOSOMAL PROTEIN S12 in A. Key antioxidant and cell wall proteins, including GLUTATHIONE PEROXIDASE (down in A and AB), ASCORBATE PEROXIDASE (down in A and AB), were differentially regulated, while MEMBRANE STEROID‐BINDING PROTEIN 2 was upregulated in A.

## 5 Discussion

The hypomethylating agent 5‐azacytidine (5‐azaC), a cytosine analogue widely used to study plant development (Dong‐ru et al. 2019; Zhong et al. 2021), interferes with DNA methyltransferases and induces genomic hypomethylation (Christman et al. 1983; Christman 2002; Cheng et al. 2019). To assess whether 5‐azaC affects bacterial viability, we performed growth assays and confirmed that *H. seropedicae* was not inhibited at the tested concentrations, in contrast to the sensitivity reported for *Escherichia coli* mutants (Previc and Richardson 1969; Guha 1984), *Bacillus subtilis* (Previc and Richardson 1969), and *Streptococcus pneumoniae* biofilm formation (Yadav et al. 2012). In maize seedlings, however, 5‐azaC induced dose‐dependent morphological effects: at 25 µM, root growth was severely impaired, whereas at 2.5 µM, milder reductions were observed; yet, shoot length and dry mass increased in the A and AB treatments, suggesting that moderate hypomethylation can enhance early shoot development and responsiveness to bacterial inoculation. Notably, bacterial treatment alone (B) promoted shoot biomass accumulation, corroborating previous reports of *H. seropedicae*'s bio‐stimulatory role in maize (Ogneva et al. 2019; Irineu et al. 2022). Although no similar effect was observed in rice (Irineu et al. 2022), this reinforces its species‐specificity. At the lowest dose (0.25 µM), no significant changes were observed, indicating minimal epigenetic reprogramming. Compared with dicots such as soybean, which showed reductions only at higher doses (100 µM) (Yadav et al. 2012; Coelho et al. 2024), maize appeared more sensitive to hypomethylation. Similar inhibitory effects have also been described in *Populus nigra* (Zhong et al. 2021). In contrast, bamboo hypomethylation promoted lateral root development (Liufu et al. 2023), whereas in *A. thaliana*, hypermethylation increased flowering despite reducing biomass (Ogneva et al. 2019). Altogether, our results highlight that epigenetic regulation and plant–microbe interactions are modulated by species‐specific developmental programmes. Among the concentrations tested, 2.5 µM emerged as optimal for studying hypomethylation in maize, as it preserved viability while revealing the interplay with *H. seropedicae*.

Global DNA methylation analysis confirmed the hypomethylating effect of 5‐azaC, while *H. seropedicae* also modulated methylation, reducing CG levels similarly to the inhibitor. Interestingly, bacteria tended to increase CHG methylation, whereas 5‐azaC was the primary driver of demethylation at this site. Comparable effects have been described in bamboo and *A. thaliana* treated with 5‐azaC or zebularine (Yao and Kovalchuk 2018; Liufu et al. 2023), while plant–microbe interactions are known to modulate methylation, including hypomethylation responses to pathogens (Pavet et al. 2006; Dowen et al. 2012; Chen et al. 2018; Hewezi et al. 2018).

At the gene level, strong modulation of epigenetic regulators was observed. DRM2 and MET were consistently downregulated in bacterial treatments (B and AB), supporting the inhibition of methylation maintenance (Ashapkin et al. 2002; Henderson et al. 2010; Liu et al. 2018). DML was repressed in B and AB but remained unaffected in A, suggesting that active demethylation is explicitly impacted by bacterial presence (Penterman et al. 2007). Among readers, MBD1 was downregulated in all treatments, while MBD7 was induced only in AB, indicating compensatory regulation (Ng et al. 2000; Lang et al. 2015).

Both SAMS repression across treatments and stable SAHH expression suggest that SAM limitation is a significant constraint on methylation (Komoto et al. 2004; Shima et al. 2017). Additionally, reduced DCL3 expression, most evident in B, suggests impaired siRNA biogenesis, affecting the RdDM regulatory pathway (Fukudome and Fukuhara 2017; Belal 2022), which is corroborated by the downregulation of CLS1‐2 in B. This scenario changed in 5‐azaC‐treated plants when CLS1‐2 and CLS3‐4 were upregulated in A and AB. Finally, the repression of ROS homologues suggests reduced active demethylation in B, A, and AB as a compensatory mechanism for hypomethylation (Williams et al. 2015; Zeng 2024).

Together, these results reveal that *H. seropedicae* and 5‐azaC interactively and distinctly reshape DNA methylation pathways in maize, reflecting a dynamic interplay between bacterial colonisation and epigenetic regulation.

Our findings demonstrate that hypomethylation induced by 5‐azaC enhances *H. seropedicae* colonisation in maize roots, as indicated by CFU counts and RT‐PCR, with higher bacterial loads observed in the AB treatments. These results support the idea that epigenetic modifications modulate plant–microbe interactions (Coelho et al. 2024). Microscopy analyses confirmed colonisation patterns consistent with previous reports (James et al. 2002; Monteiro et al. 2008), although SEM revealed fewer bacteria in the root cap of AB roots, suggesting structural alterations due to 5‐azaC treatment. Interestingly, fungal proliferation occurred only in A, but not in AB, indicating that co‐inoculation suppresses fungal growth, reinforcing the role of *H. seropedicae* in shaping microbiota composition.

Metataxonomic analysis revealed that 5‐azaC (A) increased alpha diversity and altered microbial profiles, consistent with evidence that host epigenetic changes influence microbiome assembly (Pérez‐Jaramillo et al. 2018; Zhang et al. 2023). Taxonomic shifts revealed *enrichment of Herbaspirillum* in C, B, and AB, but a reduction in A, suggesting that hypomethylation interferes with native strains, while inoculated strains remain competitive. Treatments A and AB also recruited genera associated with complex carbon metabolism, such as *Duncaniella (*Sonke and Trembath‐Reichert 2023 *)*, while *Prevotella* is recognised as a relevant component of the plant root microbiome, with the ability to degrade hemicellulose and produce organic acids such as acetate and succinate, potentially promoting nutrient cycling and plant growth (Ueki et al. 2007; Islam 2023). *Curtobacterium*, enriched in A, confirmed its role as a stress‐adapted plant growth‐promoting bacterium with biocontrol potential (Silambarasan et al. 2019; Vimal et al. 2019; Patel et al. 2022; Schillaci et al. 2022).

The presence of *Sphingomonas* and *Acinetobacter* across treatments indicates that these taxa respond more to plant physiology than to methylation status. These genera are naturally associated with the rhizosphere and root endosphere, where they contribute to plant health by promoting growth, enhancing nutrient acquisition, and improving tolerance to abiotic stresses (Luo et al. 2019; Wang et al. 2022; Castillo‐Texta et al. 2024; Sultana et al. 2024). Network analysis further highlighted treatment‐dependent shifts: B was dominated by *Herbaspirillum*, while A and AB showed denser, more complex microbial associations, including functional groups such as *Prevotella*, *Eubacterium*, and *Desulfovibrio* health (Nanninga and Gottschal 1987; Ueki et al. 2007; Li et al. 2014; Mistry et al. 2022; Islam 2023). Notably, several genera typically described in animal microbiomes (e.g., *Mucispirillum*, *Alistipes*, *Odoribacter*) (Walker et al. 2017) were enriched under hypomethylation, suggesting that relaxed methylation facilitates recruitment of atypical taxa.

Altogether, these results indicate that DNA hypomethylation reshapes root microbial communities by increasing diversity, altering exudation‐driven selection, and facilitating *the colonisation of H. seropedicae*. The enhanced connectivity observed in A and AB suggests that methylation acts as a gatekeeper of microbial recruitment, and its relaxation promotes the assembly of more complex and functionally diverse microbiomes with potential implications for plant growth, nutrient cycling, and stress tolerance.

Proteomic analysis revealed that both *H. seropedicae* inoculation and 5‐azaC‐induced hypomethylation strongly reprogramme maize root metabolism. Inoculation (B/C) enriched proteins involved in central metabolism (Ferrari et al. 2014; Nunes et al. 2021), while 5‐azaC (A/C) increased proteins linked to stress responses and phenylpropanoid biosynthesis (Liu et al. 2015; Zhang et al. 2015). Combined treatment (AB) showed intermediate modulation, suggesting synergism that favours nitrogen and carbon fixation‐related proteins.

These proteomic shifts were consistent with functional predictions from 16S data, which indicated enrichment of amino acid (lysine, arginine, glutamate) and carbon metabolism pathways in the root microbiome (Price et al. 2018; Cotton et al. 2019; Shimizu and Matsuoka 2022). In particular, microbial arginine and polyamine biosynthesis may provide substrates that enhance host phenylpropanoid metabolism and other proteins modulated by 5‐azaC (Winter et al. 2015; Paschalidis et al. 2019; Yariuchi et al. 2021). Thus, host proteome adjustments and microbial functional potential converge in complementary metabolic networks (Jing et al. 2020).

Detailed pathway analysis highlighted profound reconfiguration of carbon and amino acid metabolism. Inoculation down‐regulated PAL, a central enzyme in phenylpropanoid biosynthesis, suggesting that *H. seropedicae* is not perceived as a threat, facilitating colonisation (Dixon 2001; Vogt 2010). Concurrently, modulation of GABA metabolism and TCA cycle enzymes indicated fine‐tuning of energy flux to sustain growth and symbiosis (Bouché and Fromm 2004; Araújo et al. 2014).

By contrast, 5‐azaC disrupted methionine metabolism, reducing the precursors of SAM and reinforcing its role as a hypomethylating agent (Komoto et al. 2004; Shima et al. 2017). It also induced changes in carbohydrate fluxes, GABA metabolism, and lipid remodelling, while repressing antioxidant defences (Mittler 2002; Gill and Tuteja 2010). These findings align with the notion that DNA demethylation directly affects redox balance, cytoskeletal organisation, and stress responses (Sonenberg and Hinnebusch 2009; Mathur et al. 2014).

Combined treatments revealed complex outcomes: AB/A activated plant defences (PAL upregulation), while AB/B promoted lipid mobilisation while maintaining antioxidant repression, indicating that 5‐azaC modulates metabolic regulation in ways that favour *H. seropedicae* activity. Altogether, these results demonstrate that epigenetic modulation and bacterial inoculation converge to remodel maize root metabolism, shaping energy fluxes, amino acid biosynthesis, and other metabolic processes in ways that support colonisation, growth, and plant adaptation.

## 6 Conclusions

Our study demonstrates that DNA hypomethylation, induced by 5‐azaC, profoundly reprogrammes maize root development, epigenetic regulation, and microbial interactions. At moderate doses, 5‐azaC enhanced early shoot growth and increased responsiveness to *H. seropedicae*, while higher concentrations impaired root development, revealing maize's sensitivity to epigenetic perturbation compared with other plant species. Global and gene‐specific methylation analyses showed that both 5‐azaC and *H. seropedicae* distinctly modulate DNA methylation pathways, affecting key regulators of methylation, demethylation, and RdDM machinery.

Importantly, hypomethylation facilitated bacterial colonisation and altered root‐associated microbial communities by increasing alpha diversity, reshaping microbial networks, and recruiting functionally diverse taxa, including genera linked to carbon turnover and stress adaptation. These shifts, coupled with proteomic reprogramming, revealed convergent changes in central metabolism, amino acid biosynthesis, and phenylpropanoid pathways, highlighting synergistic interactions between host epigenetic status and microbial functional potential.

Together, our results provide evidence that DNA methylation acts as a gatekeeper of microbial recruitment and metabolic homoeostasis in maize roots. Relaxation of this control through hypomethylation promotes *H. seropedicae* colonisation, enhances microbiome complexity, and reconfigures metabolic networks, ultimately influencing plant growth, nutrient cycling, and stress resilience. These findings highlight the pivotal role of epigenetic regulation in shaping plant–microbe interactions, and they open up new avenues for leveraging epigenetic modulation to optimise beneficial symbioses in sustainable agriculture.

## Conflicts of Interest

The authors declare no conflicts of interest.

## Supporting information

Supporting File 1.

Supporting File 2.

## Data Availability

The original contributions presented in this study are included in the article/supporting material. Further inquiries can be directed to the corresponding author.
