# Supplementary material for: DNA Methylation Shapes Seed‐Borne Microbiome and Proteome Responses During Early Maize‐Beneficial Bacteria Interactions
Source: Plant Cell Environ. 2026 Apr 22;49(8):5477–96. doi: 10.1111/pce.70552 (PMC13353764; doi:10.1111/pce.70552)
Supplement: Supplementary file 1 — Supporting File 1. [file PCE-49-5477-s001.docx]

## **Supplementary Material**


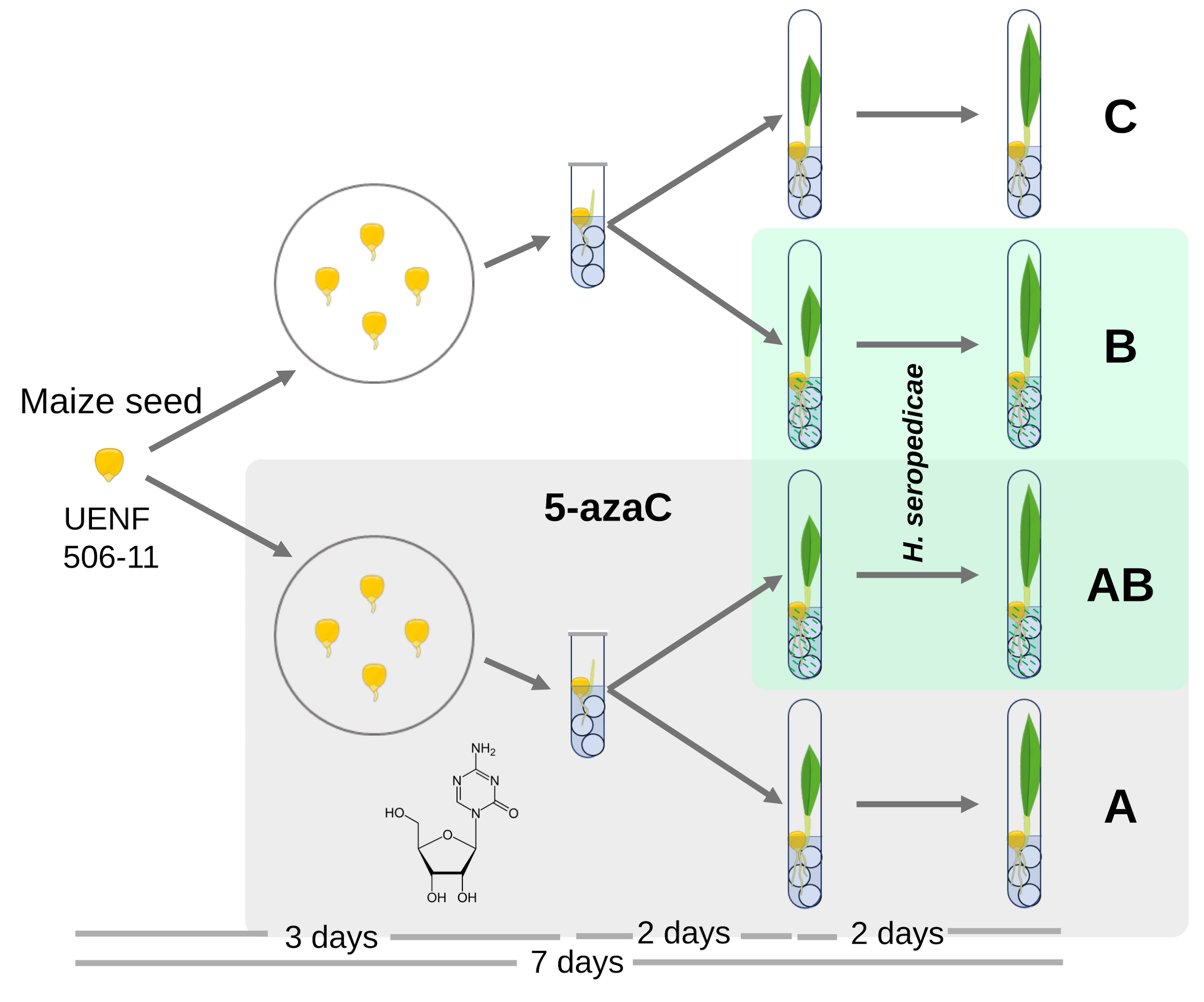


**Supplementary Figure S1.** Schematic representation of the experimental design, highlighting the different treatment conditions applied to maize seedlings.

**Table S1: Primers used for qRT-PCR analysis**

| Primer | Foward | Reverse |
| --- | --- | --- |
| TubAlfa3 | GCGCACCATCCAGTTCGT | CTGGTAGTTGATTCCGCACTTG |
| DRM2 | CAAGCACAGGGAAGTAGAGG | GATCTGTCCACTCGTCTTGAC |
| SAMS | TGTTTGGGTATGCGACTGAC | TCCATTCTTGCGAACCTCC |
| SAHH | TTCCGTCACCAAGAGCAAG | GACATCACCGTATCCGCAG |
| MBD1 | AGGAAATTAAGAACAAGAGGCAAC | CCTTGACTTTCTCGCTAATGC |
| MBD7 | GTGATTATGGGCGGTGACTAC | GGCTTTTGTACGCTGGATTTG |
| DML | CCTACCCCATACTTATTGGAA | TTGCTAAAATCGCCTCCCA |
| MET | GCCAACACATTCCGAAACG | CCCGTACAGTCCTTTCCAC |
| DCL 3 | CCTTGATAGTGGGTGTGCTAC | TCTAATCCTTCGGCTTGCTG |
| CLS1-2 | CCATCTTCCGCTGATAGTCAAG | TGCTCTCATGAACGACTTCTG |
| CLS3-4 | CGTGGGAAGCATGAATTTGTT | TTTCACGCCTTTGTCATTTGG |
| ROS1b | CCATGCTGTGACCCTCAAATG | CTCTGCAGTACAATTCTGGCAC |
| ROS1a | CCAGATGATCCCTGCCATATCTTC | GGCATCGATCGARRGTGCAGTTTC |
| Hs54C | ATTCACGCTCCCTCGACGAC | CGGGCTTGGCGTTGGTGACG |

**Table S2: ISSR primers used for cytosine methylation pattern analysis**

| Primer | Loci | Sequencia (5’-3’) | Ta (°C) |
| --- | --- | --- | --- |
| ISSR-06 | UBC- 809 | AGAGAGAGAGAGAGAGG | 48 |
| ISSR-10 | UBC- 823 | TCTCTCTCTCTCTCTCC | 48 |
| ISSR-14 | UBC- 829 | TGTGTGTGTGTGTGTGC | 53 |
| ISSR-15 | UBC- 830 | TGTGTGTGTGTGTGTGG | 52 |
| ISSR-21 | UBC- 841 | GAGAGAGAGAGAGAGAYC | 48 |
| ISSR-25 | UBC- 847 | CACACACACACACACARC | 53 |
| ISSR-31 | UBC- 859 | TGTGTGTGTGTGTGTGRC | 54 |

**
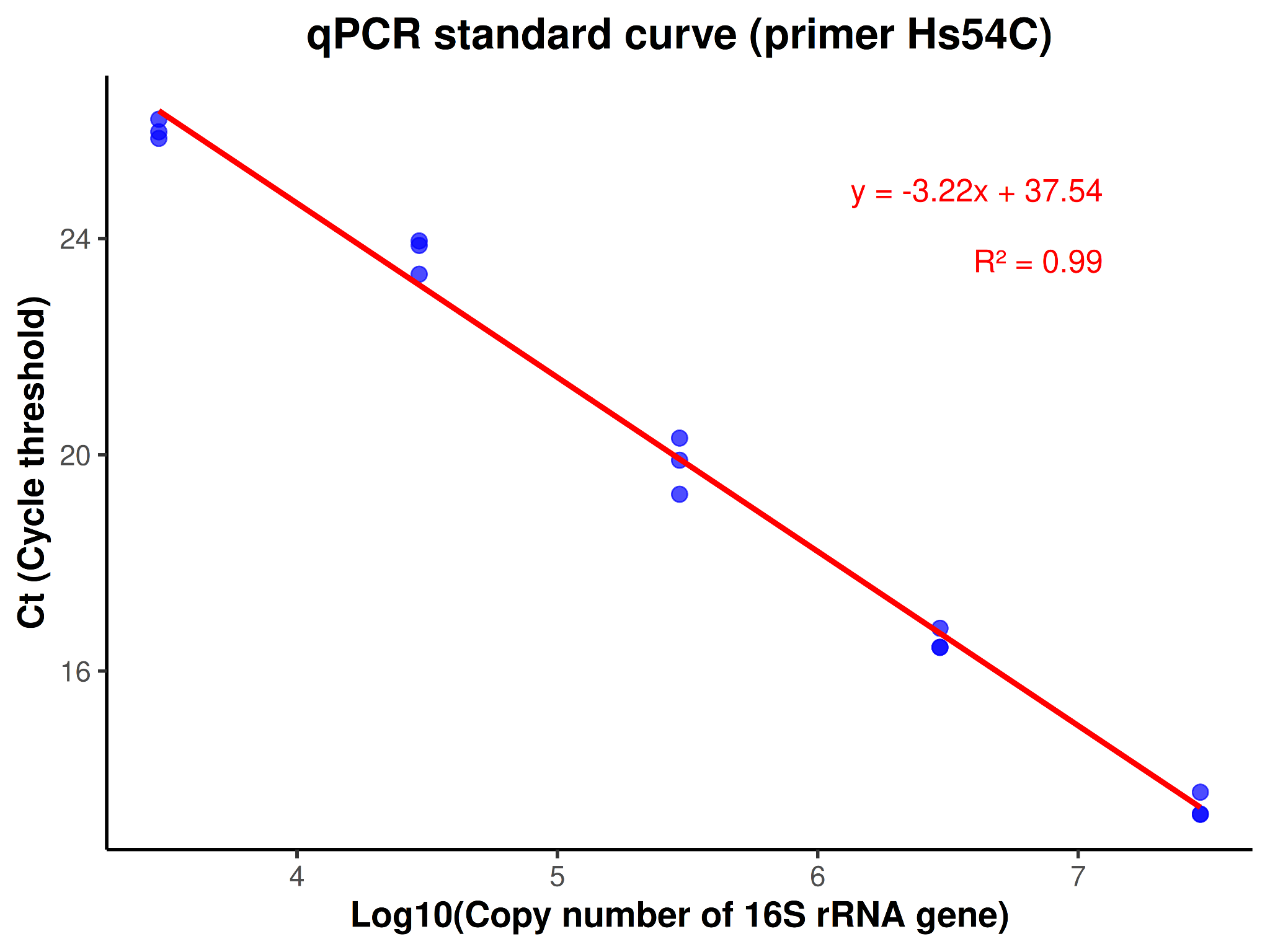
**

**Supplementary Figure S2. qPCR standard curve using primers for the HS54C region with *H seropedicae*.** Serial dilutions of *H. seropedicae* genomic DNA were amplified. Ct values are plotted against the logarithm (Log10) of the copy number. Regression equations and coefficients of determination (R²) are indicated for the curve.

**Table S3: Strategy used to interpret different banding patterns and quantify genome methylation percentage**

| Banding Pattern | Restriction Sites | Interpretation |
| --- | --- | --- |
| DNA/ Hpa II/ MspI |  |  |
| 1/1/1 | ^5^'CCGG3' | Unmethylated cytosines |
| 1/0/1 | ^5^'CmCGG^3^' | Fully methylated CG sites |
| 1/1/0 | ^5'm^CCGG^3'^ | Hemimethylation at CHG sites |
| 1/0/0 | ^5'm^C^m^CGG^3^  ^' 5'^CCNG^3^' | Methylation on both cytosines or unknown mutation |


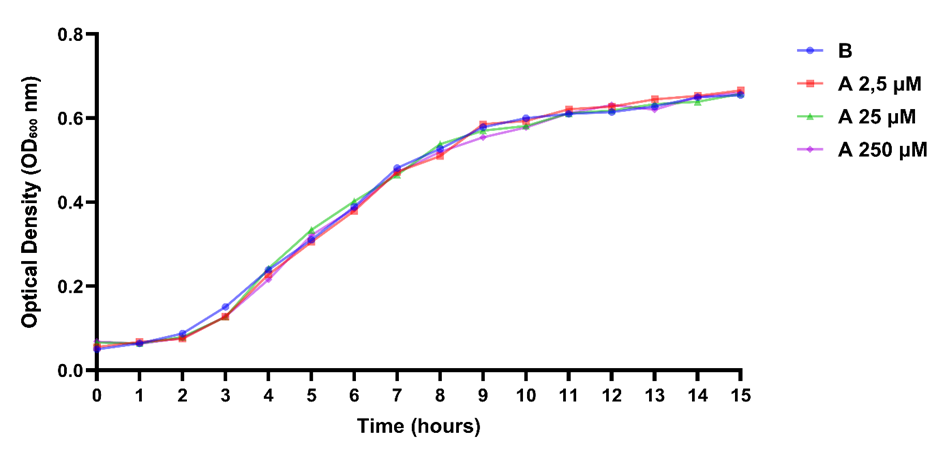


**Supplementary Figure S3. Growth curves of *H. seropedicae* treated with 5-azaC (A)**. Bacteria were exposed to 2.5 μM, 25 μM, and 250 μM of 5-azaC, as well as a control group without treatment. No significant differences (p > 0.05) were detected using ANOVA analysis.


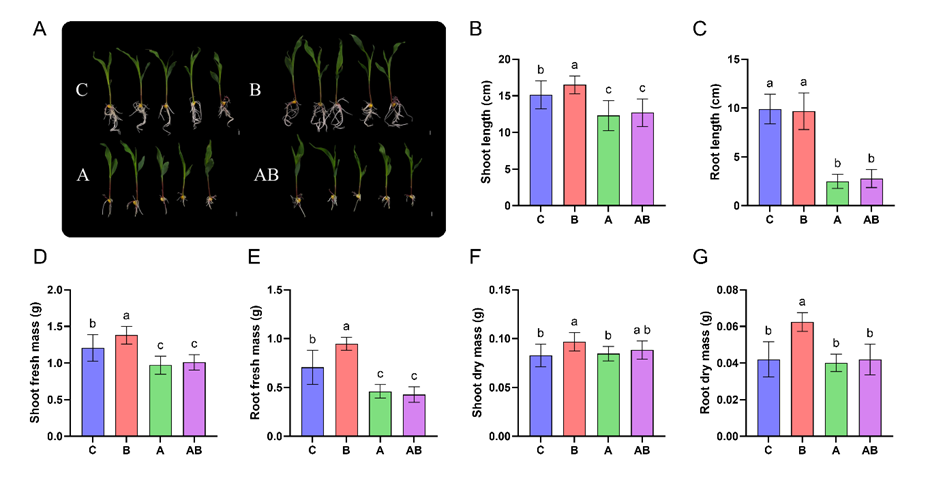


**Supplementary Figure S4 Effect of the methylation inhibitor (5-azaC) on maize seedlings development at 7 DAE. Treatments included 25 µM 5-azaC and inoculation with H. seropedicae for 24 HAI.** (A) Image showing the effect of the compound on seedling growth (Scale bar = 1 cm). (B, D, F) Measurements of shoot length, fresh mass, and dry mass, respectively. (C, E, G) Measurements of root length, fresh mass, and dry mass, respectively. Letters (a, b, c, d) indicate significant differences between treatments based on Tukey's test. DAE – Days After Emergence; HAI – Hours After Inoculation. In the graphs, the labels C, B, A, and AB correspond to Control, Bacteria, 5-azaC, and 5-azaC + Bacteria, respectively


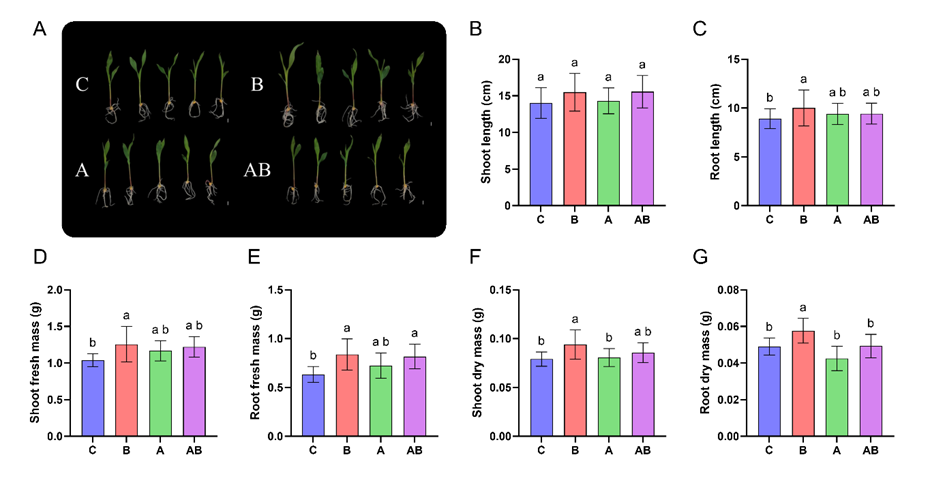


**Supplementary Figure S5 Effect of the methylation inhibitor (5-azaC) on maize seedlings development at 7 DAE. Treatments included 0,25 µM 5-azaC and inoculation with H. seropedicae for 24 HAI.** (A) Image showing the effect of the compound on seedling growth (Scale bar = 1 cm). (B, D, F) Measurements of shoot length, fresh mass, and dry mass, respectively. (C, E, G) Measurements of root length, fresh mass, and dry mass, respectively. Letters (a, b, c, d) indicate significant differences between treatments based on Tukey's test. DAE – Days After Emergence; HAI – Hours After Inoculation. In the graphs, the labels C, B, A, and AB correspond to Control, Bacteria, 5-azaC, and 5-azaC + Bacteria, respectively.


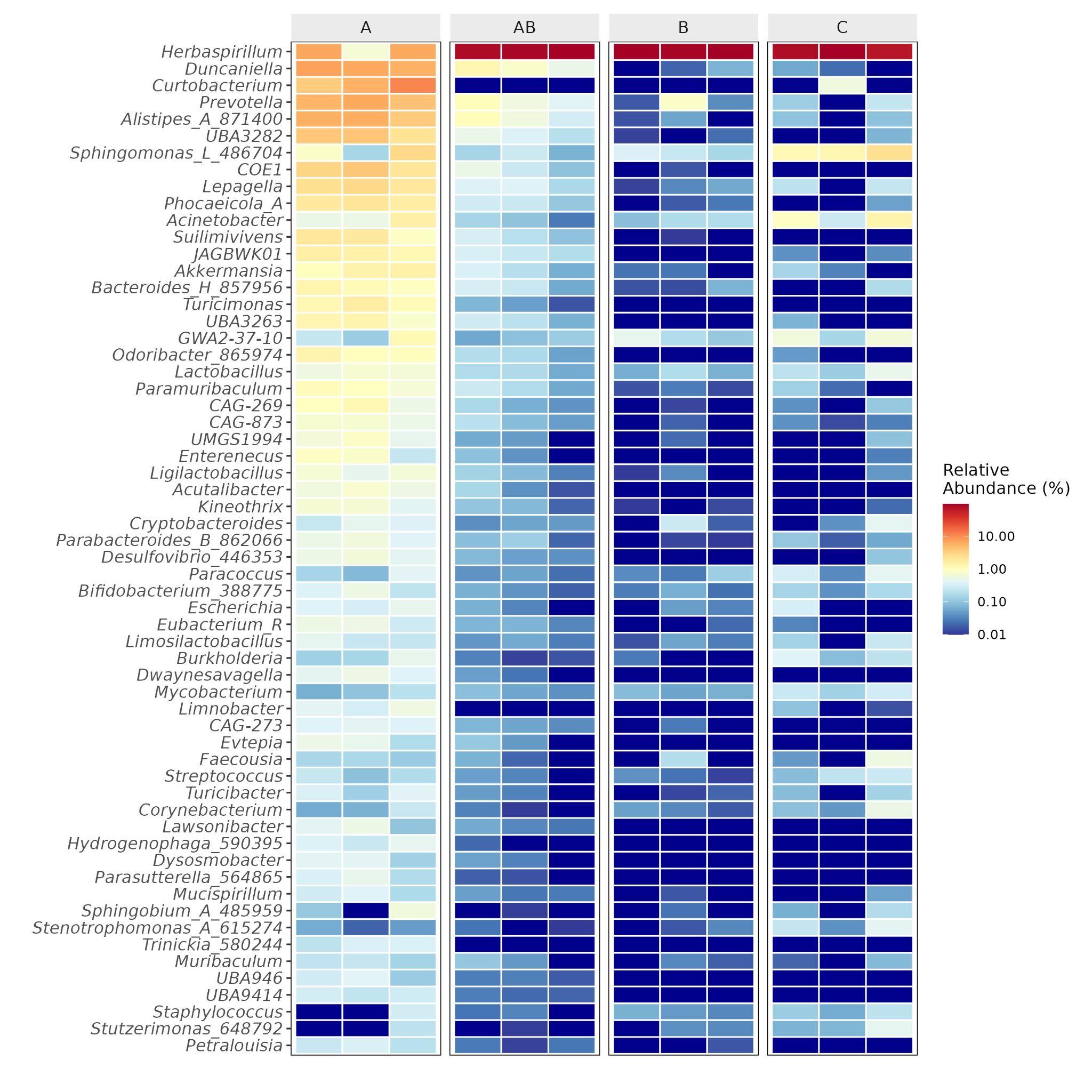


**Supplementary Figure S6.** Heatmap of the relative abundance of the most prevalent bacterial genera across different samples. Treatments are represented as follows: C (Control), B (Bacteria), A (5-azaC), and AB (5-azaC + Bacteria).


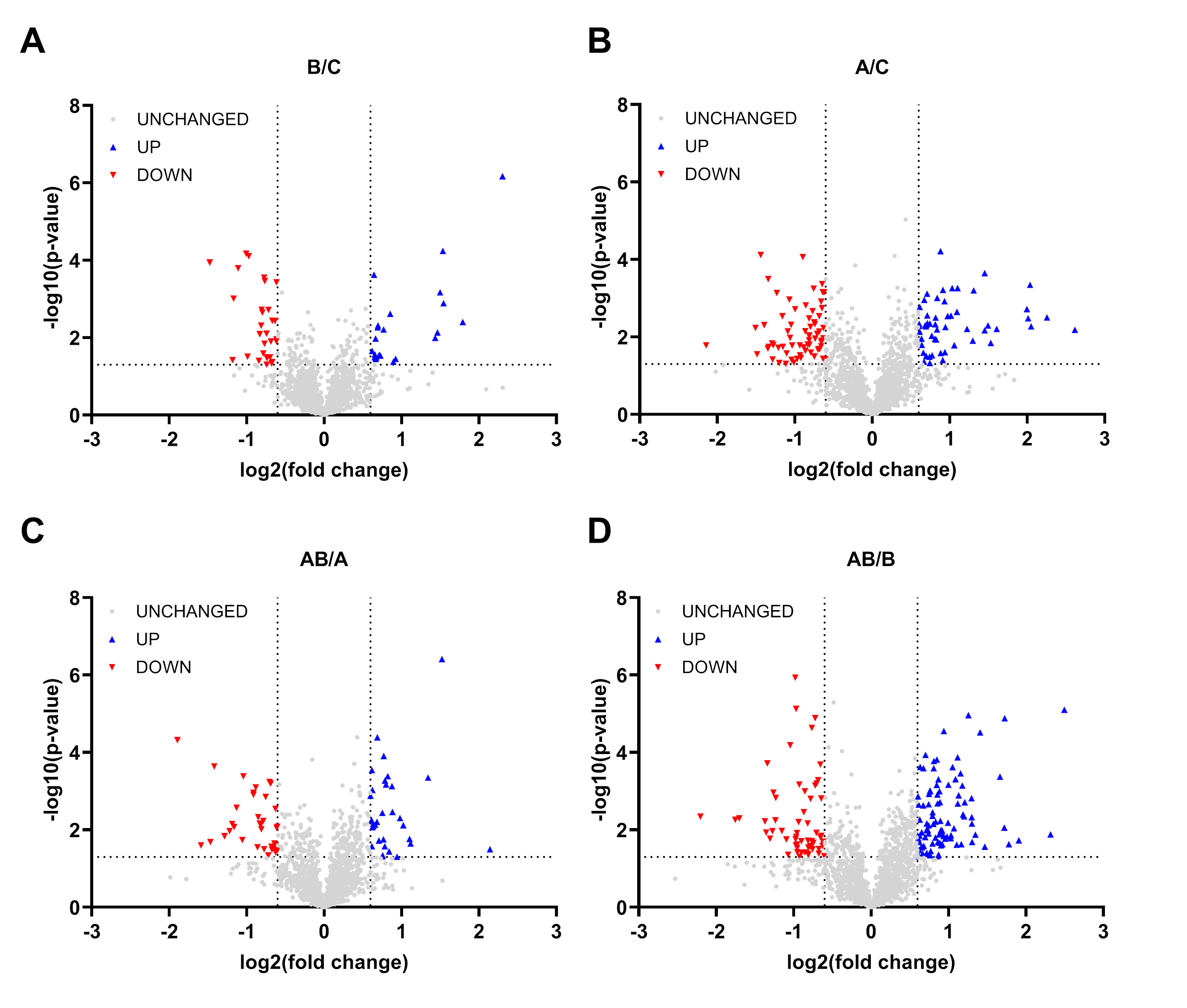


**Supplementary Figure S7. Differentially accumulated proteins in maize seedling roots are treated with the DNA methylation inhibitor 5-azaC (2.5 µM) for 7 DAI and inoculated with *H. seropedicae* for 48 HAI.** Volcano plot of differentially accumulated proteins. Blue dots: up-accumulated proteins (log2 FC ≥ 0.5, P ≤ 0.05). Red dots: down-accumulated proteins (log2 FC ≤ -0.5, P ≤ 0.05). (A) Comparison B/C. (B) Comparison A/C. (C) Comparison AB/A. (D) Comparison AB/B.
